# British Workers in Foreign Countries: Hospital Life in Kashmir

**Published:** 1914-01-10

**Authors:** 


### BRITISH WORKERS IN FOREIGN COUNTRIES.

### Hospital Life in Kashmir.

In Great Britain institutional affairs, be they administrative, financial, medical, surgical, or nursing, proceed along lines which are in broad essentials fixed. But in countries which, as far as institutions for the sick are concerned, are new, the capable and energetic institutional manager may mould his hospital into any shape he thinks fit. Very often he is secretary, superintendent, steward, physician, surgeon, and even treasurer all rolled into one; and when an able man of commanding personality is found in such a position of responsibility it is not surprising that committees, hundreds or thousands of miles from the scene of labour, should leave him a free hand.

Missionary hospitals in many parts of the world supply instances of these points. The fame of the brothers Cook and their hospital in Uganda, for instance, is spread far and wide through Africa; and among notable cases in Asia, the medical missionaries of the Church Missionary Society in India and China are deserving of particular notice. During the last two or three years death has taken a heavy toll amongst these workers. But the work goes on, and we welcome a new book by one of the best known of this gallant band,* describing his work and play in the remote and curious province of Kashmir.

It was, apparently, chance which first took Mr. Neve to the East; for he was attracted by Central Africa, and on attaining his medical qualification applied to the Church Missionary Society for work to do in that continent. Just at that moment it so happened that two doctors in succession had broken down in Kashmir; so the Society requested their new recruit to take up the medical mission thus left vacant. This was in 1882, when pathways were blocked by landslides, and primitive bridges often washed away by floods. In 1886 Mr. Neve was joined by his brother, Dr. Ernest Neve, and both have been up to the hilt in work ever since, save for furloughs home. In twelve years the brothers rebuilt the hospital (at Srinagar) completely, erecting a series of ward blocks which contained ultimately 140 beds. To-day there is a commodious out-patient building with three operation-rooms, a bacteriological laboratory, and consulting-rooms and *x*-ray outfit.

At special seasons, chiefly religious festivals of the (Mohammedan) Kashmiris, the press of work at the hospital becomes tremendous. Twenty major, fifty minor, operations, and three to five hundred out-patient consultations represent a very fair day's work for three medical men to cope with! A special leprosy hospital has been started, and now accommodates ninety lepers.

Mr. Neve is an enthusiastic Christian missionary, as well as a hospital organiser and a surgeon. But he has his own ideas of how Christian missions should approach the heathen. They should not, he says emphatically, aim at the reproduction among Indians of the special forms of denominational Christianity with which we are familiar in Europe. The Central Committee in India, of all non-Roman missions, has outlined a much wider policy. A federation of all the existing ecclesiastical bodies is looked forward to on the basis of some exceedingly simple formula embodying the essential doctrine of the Christian religion. India, in fact, should have its own National Church, according to this author; an ideal which is still a long distance from achievement. Nor can it be forgotten in this connection that the so-called Ethiopian movement in South Africa, originally a purely religious organisation (in fact an African Church), speedily developed into a seditious and revolutionary political society, and was deeply involved in the Zulu rebellion of 1906.

* *Thirty Years in Kashmir.* By Arthur Neve, F.R.C.S.Ed. Edward Arnold. Pp. 316. Price 12s. 6d. net.

